# Monocyte anisocytosis corresponds with increasing severity of COVID-19 in children

**DOI:** 10.3389/fped.2023.1177048

**Published:** 2023-06-23

**Authors:** Abigail S. Kane, Brittany P. Boribong, Maggie Loiselle, Anagha P. Chitnis, Hector Chavez, Lyle L. Moldawer, Shawn D. Larson, Oluwakemi Badaki-Makun, Daniel Irimia, Lael M. Yonker

**Affiliations:** ^1^ Department of Pediatrics, Massachusetts General Hospital, Boston, MA, United States; ^2^ Mucosal Immunology and Biology Research Center, Massachusetts General Hospital, Boston, MA, United States; ^3^ Department of Pediatrics, Harvard Medical School, Boston, MA, United States; ^4^ Department of Pediatrics, Jackson Memorial Hospital, Miami, FL, United States; ^5^ Department of Pediatric Emergency Medicine, Holtz Children’s Hospital, Miami, FL, United States; ^6^ Department of Surgery, University of Florida, Gainesville, FL, United States; ^7^ Department of Pediatrics, Johns Hopkins School of Medicine, Baltimore, MD, United States; ^8^ Center for Data Science in Emergency Medicine, Johns Hopkins School of Medicine, Baltimore, MD, United States; ^9^ Department of Surgery, Center for Engineering in Medicine, Massachusetts General Hospital, Boston, MA, United States; ^10^ Department of Surgery, Shriners Burn Hospital, Boston, MA, United States

**Keywords:** monocyte anisocytosis, pediatric COVID-19, multisystem inflammatory syndrome, SARS-COV-2 infection, monocyte distribution width

## Abstract

**Introduction:**

Although SARS-CoV-2 infection can lead to severe COVID-19 in children, the role of biomarkers for assessing the risk of progression to severe disease is not well established in the pediatric population. Given the differences in monocyte signatures associated with worsening COVID-19 in adults, we aimed to determine whether monocyte anisocytosis early in the infectious course would correspond with increasing severity of COVID-19 in children.

**Methods:**

We performed a multicenter retrospective study of 215 children with SARS-CoV-2 infection, Multisystem Inflammatory Syndrome in Children (MIS-C), convalescent COVID-19, and healthy age-matched controls to determine whether monocyte anisocytosis, quantified by monocyte distribution width (MDW) on complete blood count, was associated with increasing severity of COVID-19. We performed exploratory analyses to identify other hematologic parameters in the inflammatory signature of pediatric SARS-CoV-2 infection and determine the most effective combination of markers for assessing COVID-19 severity in children.

**Results:**

Monocyte anisocytosis increases with COVID-19 severity and need for hospitalization. Although other inflammatory markers such as lymphocyte count, neutrophil/lymphocyte ratio, C-reactive protein, and cytokines correlate with disease severity, these parameters were not as sensitive as MDW for identifying severe disease in children. An MDW threshold of 23 offers a sensitive marker for severe pediatric COVID-19, with improved accuracy when assessed in combination with other hematologic parameters.

**Conclusion:**

Monocyte anisocytosis corresponds with shifting hematologic profiles and inflammatory markers in children with COVID-19, and MDW serves as a clinically accessible biomarker for severe COVID-19 in children.

## Introduction

Throughout the course of the COVID-19 pandemic, hospitalization and mortality rates from SARS-CoV-2 infection have been markedly lower in children relative to adults. These differences in disease severity outcomes are multifactorial, but largely reflect age-related differences in immune responses against SARS-CoV-2 (1–5). Despite relative protection, COVID-19 emerged as a leading cause of pediatric death during the height of the pandemic, resulting in more annual deaths in children than any other vaccine-preventable disease, including influenza (6). Therefore, the development of biomarkers that aid in early recognition of severe COVID-19 in children is urgently needed.

Risk assessment tools for determining COVID-19 severity and mortality have primarily been studied in adults (7). Complete blood count (CBC) parameters, such as lymphopenia and neutrophilia are often used to predict disease progression, and non-specific markers of inflammation, including procalcitonin (PCT), lactate dehydrogenase (LDH), C-reactive protein (CRP), erythrocyte sedimentation rate (ESR) and neutrophil-lymphocyte ratio (NLR) have been described in COVID-19 as predictors of admission to adult intensive care unit (8). Although these are practical tools in adults, there is insufficient data to accurately portray the clinical utility of these parameters when used alone or in combination to predict severe disease in children.

Monocytes play an important role in distinguishing disease severity in SARS-CoV-2 infection (9), likely due to their involvement in antigen detection and presentation, and T cell activation. However, capturing monocytic activation is challenging. Absolute monocyte counts may not reflect shifts in monocyte populations, and while monocyte-derived cytokines may correlate with disease severity, cytokine panels are not routinely available for clinical decision-making. Even the cytokine interleukin-6 (IL-6), which has been shown to aid in risk stratification among adults with COVID-19, is not routinely ordered in pediatric cases (10). Monocyte anisocytosis, which can be quantified by monocyte distribution width (MDW) on a hematology profile, has been shown to correspond with immune activation in sepsis (11–16), severe COVID-19 in adults (17) and more recently, Multisystem Inflammatory Syndrome in Children (MIS-C) (18). We therefore hypothesized that this hematologic parameter could capture the phenotypic changes that result from monocytic activation in response to SARS-CoV-2 infection in children, and correspond with clinical severity.

Here, we performed a retrospective multicenter clinical study of 215 pediatric patients infected with SARS-CoV-2 and healthy, age-matched controls to determine whether monocyte anisocytosis was associated with increasing severity of COVID-19 in children. We subsequently performed additional exploratory analyses to identify other hematologic parameters in the inflammatory signature of pediatric SARS-CoV-2 infection, and determine the most effective combination of parameters for assessing COVID-19 severity in children.

## Materials and methods

### Subject selection and study design

Blood specimens were collected from pediatric patients 21 years of age and younger who presented for medical care between April 2020 and September 2021 at the following sites: Massachusetts General Hospital, Boston, MA; Johns Hopkins University Hospital, Baltimore, MD; University of Florida Health Science Center, Gainesville, FL; Jackson Memorial Hospital, Miami, FL (MGB IRB 2020P002961). Additionally, blood samples from subjects who were enrolled in the Pediatric COVID-19 Biorepository at Massachusetts General Hospital (MGB IRB 2020P00955) between April 2020 and August 2022 were used for analysis. Informed consent from participants and/or legal guardians was obtained prior to enrollment and sample collection, and assent was obtained from patients between 7 and 18 years of age. All study procedures were performed following the Mass General Brigham Institutional Review Board guidelines and regulations.

Demographic and clinical information were extracted from medical records and stored using REDCap electronic database. Patients were selected based on clinical status, and separated into the following cohorts: healthy controls, COVID-19, MIS-C, COVID-recovered. Healthy patients had no prior history of SARS-CoV-2 infection or significant comorbid disease and were asymptomatic at time of collection. Patients with COVID-19 were further categorized by disease severity per WHO criteria (19): subjects with mild disease were symptomatic without evidence of viral pneumonia or hypoxia; moderate COVID-19 was defined by clinical or radiographic evidence of non-severe pneumonia (i.e., fever, cough, dyspnea, tachypnea, SpO_2_ ≥ 90% on room air); severe disease included children with one or more clinical signs of severe pneumonia: severe respiratory distress, including tachypnea >30 breaths per minute in adolescents, severe chest wall indrawing, grunting or central cyanosis, SpO_2_ < 90% on room air, inability to feed or drink, altered mental status, or convulsions. Patients with MIS-C met CDC diagnostic criteria (20), and those classified as COVID-recovered had documented history of SARS-CoV-2 infection by PCR or antigen test and were asymptomatic and non-infectious at the time of sample collection. Retrospective analysis of data collected by consecutive sampling was reported following the Standards for Reporting Diagnostic Accuracy Studies (STARD) guidelines (21).

### Hematologic analysis and cytokine profiling

Venous blood was collected from patients across all cohorts in di-potassium ethylenediaminetetraacetic acid (K_2_ EDTA) anticoagulant phlebotomy tubes (Becton, Dickinson and Company, Franklin Lakes, NJ). Complete blood counts including monocyte distribution width were obtained within two hours of sample collection using the DxH900 Hematology Analyzer (Beckman Coulter, Brea, CA). Monocyte volume was obtained and used to automatically calculate monocyte distribution width (MDW) (14, 18). After collection and hematologic analysis, whole blood was centrifuged at 1,000 g for 10 min with breaks activated, and plasma was aliquoted and stored at −80°C (PMID: 32818214). Additional inflammatory biomarkers, including C-reactive protein (CRP) and erythrocyte sedimentation rate (ESR), were extracted from medical records, and used for analysis when obtained within 72 h of MDW.

Multiplex cytokine analysis was performed using CodePlex Secretome Human Innate Immune Panel (IsoPlexis, Inc., Branford, CT), following the manufacturer’s instructions. In brief, plasma was thawed on ice and CodePlex chips were thawed at room temperature for 60–75 min. Plasma samples were mixed prior to loading 5.5 µl onto duplicate wells of the CodePlex chip. Two percent bovine serum albumin (BSA) was used for background control measurements. CodePlex chips were placed in the IsoLight instrument and analyzed by IsoSpeak software, which provided quantitative measurements for the following cytokines: EGF, GM-CSF, Granzyme B, INF- γ, IL-1β, IL-4, IL-6, IL-7, IL-8, IL-10, IL-15, IP-10, MCP-1, MIP-1α, MIP-1β, PDGF-BB, sCD137, TNF-α, and VEGF.

### Statistical analysis

Statistical analysis and figure design were completed using Prism GraphPad (Version 9.5.0) and IBM SPSS Statistics (Version 24). Single MDW outliers were identified by Grubb’s outlier test and removed prior to analysis. Non-parametric data was analyzed using Kruskal-Wallis and Dunn’s multiple comparisons tests, whereas analysis of normally distributed data was done using one-way-ANOVA parametric test and Tukey’s multiple comparisons test, or unpaired t-test. Raw data was analyzed and displayed either in linear or log scale, as noted. Simple linear regression was used to determine change in MDW over time. Receiver operator curves (ROC) were obtained to determine the sensitivity and specificity of laboratory tests for distinguishing disease severity. Cut-off values were determined based on maximum sensitivity and specificity, allowing for maximum detection of severe cases of COVID-19, and the overall diagnostic effectiveness of the tests was estimated using Youden’s J statistic. Non-parametric measures of rank correlation were obtained using Spearman’s rank correlation coefficient*,* and categorical values were assessed using Fisher’s exact test.

## Results

**Figure 1:**
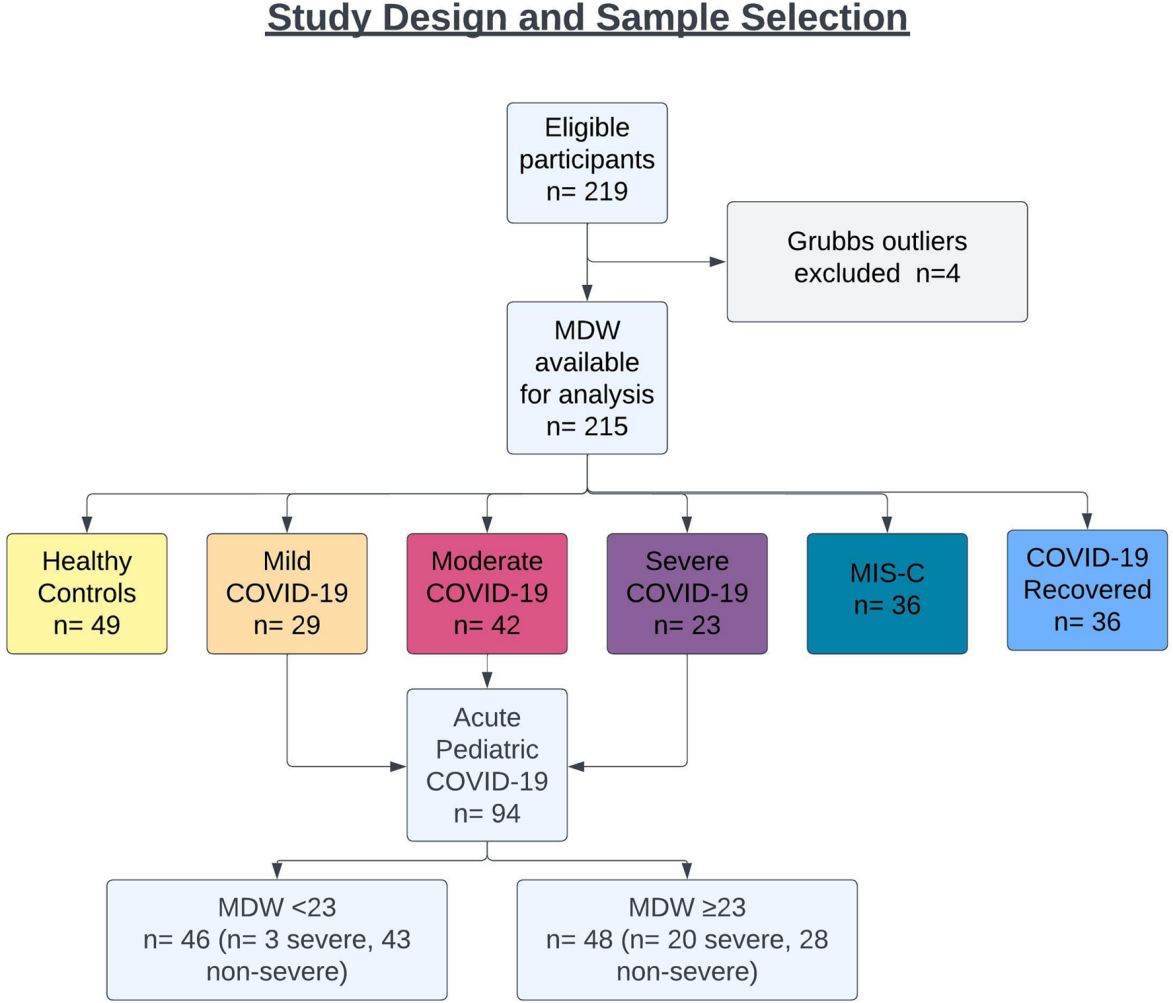
STARD flow diagram of study design, sample collection and cohort distribution.

Blood specimens were prospectively collected from 897 children presenting for medical care across four medical centers from April 2020 through August 2022. To test whether hematologic parameters could be used to determine disease severity in children infected with SARS-CoV-2, we specifically focused on blood samples from 215 pediatric patients, including children presenting with acute COVID-19 (*n* = 94, 44%), children who progressed to MIS-C after SARS-CoV-2 infection (*n* = 36, 17%), asymptomatic children recovered from prior SARS-CoV-2 infection (*n* = 36, 17%), and age-matched, uninfected healthy subjects (*n* = 49, 23%) (Figure 1). Nearly half of participants were female (47%), and mean age at sample collection was 12 years (range: 0.2–21 years). Eighty-three (39%) patients were of Hispanic or Latino ethnicity, and racial distribution, when specified, was as follows: Asian (*n* = 8, 4%), Black or African American (*n* = 38, 18%), and White (*n* = 88, 41%). COVID-19 infection was confirmed by detection of SARS-CoV-2 via nasopharyngeal or anterior nasal PCR. COVID-19 patients were classified by WHO disease severity criteria as mild (symptomatic without pneumonia), moderate (clinical or radiographic evidence of pneumonia without hypoxia), and severe (severe pneumonia, hypoxia, or respiratory distress) (19). Demographic information for healthy controls and SARS-CoV-2-infected cohorts are listed in Table 1.

**Table 1:** Patient demographics.

	All children	Demographic information by cohort					
	(*N* = 215)	Healthy	Mild COVID-19	Moderate COVID-19	Severe COVID-19	MIS-C	COVID-Recovered
		(*n* = 49)	(*n* = 29)	(*n* = 42)	(*n* = 23)	(*n* = 36)	(*n* = 36)
Age at enrollment, mean years (min, max)	12 (0.2, 21)	14 (6, 19)	10 (0.8, 21)	12 (0.2, 21)	14 (0.8, 21)	10 (1,21)	12 (0.17, 20)
Male, *n* (%)	115 (53)	21 (43)	13 (45)	26 (62)	15 (65)	23 (64)	17 (47)
Hispanic, *n* (%)	83 (39)	16 (33)	15 (52)	17 (40)	7 (30)	11 (31)	17 (47)
Race, *n* (%)							
Asian	8 (4)	3 (6)	1 (3)	2 (5)	0 (0)	1 (3)	1 (3)
Biracial	5 (2)	3 (6)	0 (0)	0 (0)	0 (0)	2 (6)	0 (0)
Black or African American	19 (9)	3 (6)	2 (7)	12 (29)	4 (17)	15 (42)	2 (6)
Other	68 (32)	17 (35)	13 (45)	11 (26)	7 (30)	5 (14)	15 (42)
Unknown or Unreported	8 (4)	3 (6)	1 (3)	1 (2)	0 (0)	1 (3)	2 (6)
White or Caucasian	88 (41)	20 (41)	12 (41)	16 (38)	12 (52)	12 (33)	16 (44)
Median time between SARS-CoV-2 detection by PCR of nasal swab & sample collection, days (range)	n/a	n/a	0 (0–14)	1.5 (0–10)	5 (0–11)	n/a	188 (24–290)
Median time between MIS-C hospitalization to sample collection, days (range)	n/a	n/a	n/a	n/a	n/a	1 (0–10)	n/a

Demographic information for each cohort.

Hematology parameters were measured directly from whole blood collected in an EDTA phlebotomy tube by hematology analyzer. Blood was collected from children with COVID-19 upon presentation to urgent care clinic or during hospitalization; the median time from diagnosis of COVID-19 by PCR to blood collection was 1 day (range: 0–14 days). Children with MIS-C also provided blood samples for analysis at time of hospitalization (median time from admission for MIS-C: 1 day, range: 0–10 days). Median time from SARS-CoV-2 infection to sample collection in COVID-19-recovered patients was 188 days (range: 24–290 days) (Table 1). Clinical descriptions of maximal level of care and need for supplemental respiratory support for children with acute COVID-19 are shown in Supplementary Table S1. While respiratory symptoms and general symptoms of fever and fatigue were common in both mild/moderate and severe COVID-19, cardiovascular, neurologic and musculoskeletal symptoms were less common; however, gastrointestinal symptoms were significanty increased in children with severe COVID-19 (Table 2; Fisher’s exact test *p =* 0.01).

**Table 2:** Symptoms of acute pediatric SARS-CoV-2 infection.

Symptoms by Organ System	Mild/Moderate COVID-19	Severe COVID-19	*p=*
	(*n* = 71)	(*n* = 23)	
**General, *n* (%)**	49 (69)	18 (78)	0.199
Fever	44 (62)	18 (78)	** **
Fatigue	16 (23)	5 (22)	** **
**Respiratory, *n* (%)**	58 (82)	21 (91)	0.096
Nasal Congestion	24 (34)	6 (26)	** **
Rhinorrhea	10 (14)	1 (4)	** **
Sore Throat	13 (18)	4 (17)	** **
Cough	39 (55)	15 (65)	** **
Shortness of Breath	22 (31)	18 (78)	** **
Wheezing	5 (7)	2 (9)	** **
Tachypnea	2 (3)	8 (35)	** **
**Gastrointestinal, *n* (%)**	40 (56)	17 (74)	0.011
Abdominal Pain	13 (18)	3 (13)	** **
Nausea/Vomiting	18 (25)	11 (48)	** **
Diarrhea	11 (15)	4 (17)	** **
Decreased PO	26 (37)	10 (43)	** **
**Cardiovascular, *n* (%)**	20 (28)	8 (35)	0.361
Chest Pain	11 (15)	7 (30)	** **
Palpitations	3 (4)	0 (0)	** **
**Neurologic, *n* (%)**	26 (37)	6 (26)	0.127
Headache	19 (26)	5 (22)	** **
Ageusia	3 (4)	1 (4)	** **
Anosmia	7 (10)	3 (13)	** **
**Musculoskeletal, *n* (%)**	18 (25)	5 (22)	0.739
Myalgia	16 (23)	5 (22)	** **
Arthralgia	2 (3)	0 (0)	** **

Symptom burden by organ involvement in children with non-severe (mild/moderate) and severe COVID-19. Analysis by Fisher’s exact test; *p* < 0.05 was considered significant.

Bold values represent data for the organ system involved.

### Monocyte anisocytosis is associated with increasing COVID-19 severity

**Figure 2:**
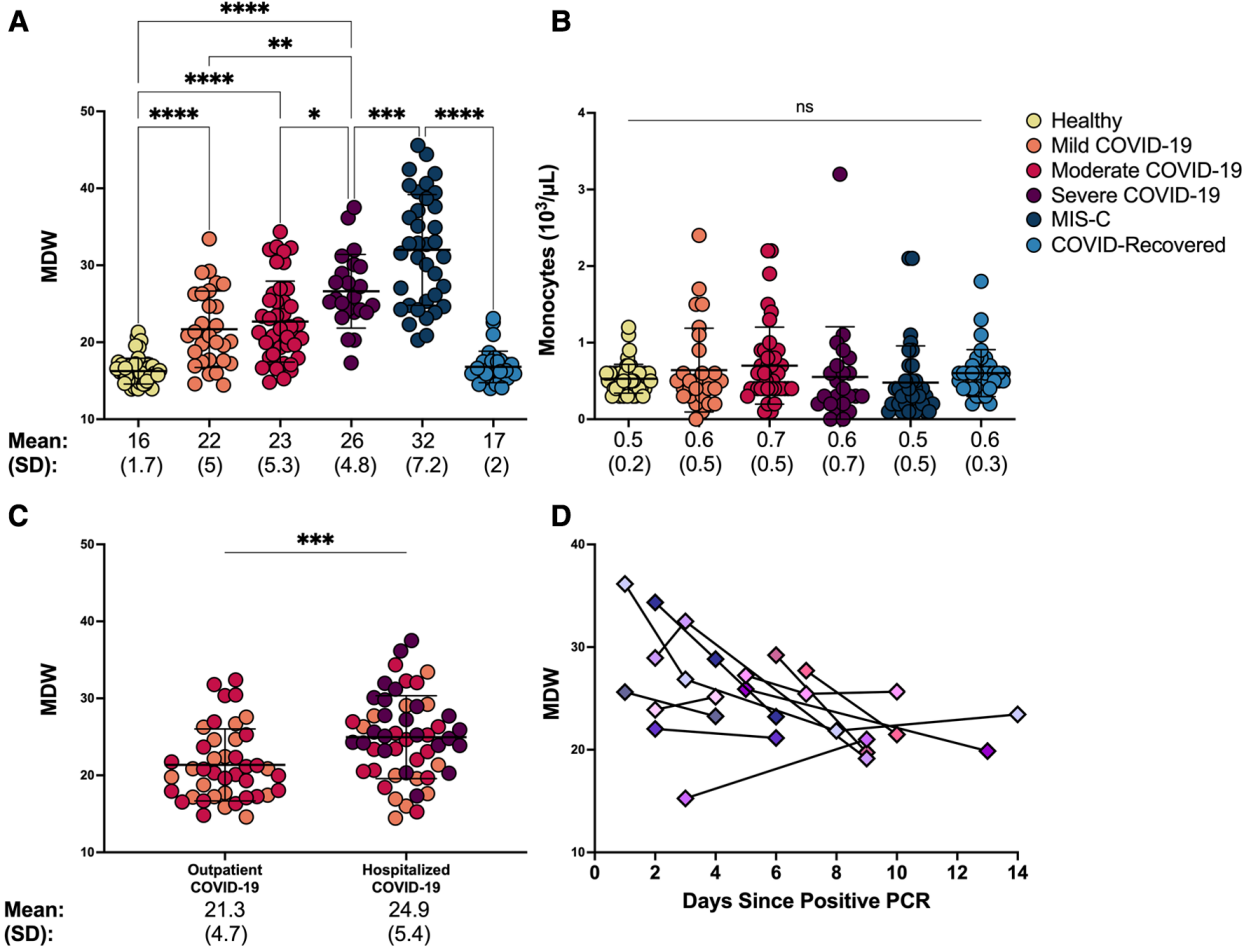
Monocyte anisocytosis reflects COVID-19 severity and need for hospitalization and improves over time. (**A**) Monocyte distribution width (MDW) and (**B**) absolute monocyte count were quantified in blood samples from healthy controls, patients with mild, moderate and severe COVID-19, MIS-C, and asymptomatic children after recovery from COVID-19. Statistical analyses were done by one-way ANOVA and Tukey’s multiple comparisons test. **** *p *< 0.0001, ***p < 0.001, **p < 0.01, *p < 0.05, ns = not significant. (**C**) MDW in children with COVID-19 was compared based on hospitalization status (outpatient *n* = 42; hospitalized *n* = 52) using unpaired t-test. ***p < 0.001. (**D**) MDW trend over the first 14 days of infection in 11 hospitalized patients with COVID-19.

Because monocytes play a key role in driving COVID-19 severity in adults (9), we tested whether monocyte parameters would correspond with disease severity in children. We found that MDW increased significantly with worsening severity of SARS-CoV-2 related illness in our cohort (Figure 2A; ANOVA *p *< 0.0001, Supplementary Table S2). While healthy children displayed an MDW of 16 ± 1.7, consistent with age-specific normative values (22), MDW increased significantly in children with mild COVID-19 (MDW mean 22 ± 5), children with moderate COVID-19 (MDW mean 23 ± 5.3), and children with severe COVID-19 (MDW mean 26 ± 4.8) (Figure 2A). Importantly, MDW in severe COVID-19 was significantly increased as compared to children with mild or moderate disease (Figure 2A: ANOVA *p *< 0.002 and *p *< 0.01 respectively). Children who developed the hyperinflammatory post-COVID-19 illness, MIS-C, displayed the highest MDW (mean: 32 ± 7.2), whereas those who recovered following COVID-19 displayed a restoration of normative MDW values (MDW mean 17 ± 2, Figure 2A). Notably, total monocyte counts did not distinguish between severity of disease (Figure 2B). Altogether, these findings demonstrate that MDW is increased in children with acute COVID-19, especially severe COVID-19, while children with post-infectious MIS-C display the highest MDW values.

To further understand clinical utility of monocyte anisocytosis in determining COVID-19 severity in children beyond what is reflected by the WHO severity scale, we sought to determine whether MDW corresponded with the need for hospitalization, given that children may be hospitalized for other non-respiratory COVID-19 symptoms such as lethargy, poor feeding and dehydration, worsening of underlying medical conditions. Importantly, MDW was significantly increased in those requiring hospitalization as compared to those who were discharged home, regardless of WHO severity rating (Figure 2C, unpairerd *t*-test *p *= 0.0009).

We then sought to determine whether monocyte anisocytosis correlated with clinical improvement by assessing serial MDW measurements from a subset of hospitalized children with COVID-19 (*N* = 11; PICU *n* = 7, ward *n* = 4). Repeat samples were obtained for up to two weeks into hospitalization when patients were required to undergo clinical phlebotomy. Individual MDW values over time are displayed in Figure 2D, with MDW declining by a median 0.79 MDW units/day (interquartile range: −2.1, −0.22). We found that compared to initial MDW assessments, monocyte anisocytosis improved over time with resolution of infection, (Supplementary Figure S1, ANOVA *p *= 0.0004), similar to reports in adults (17, 23). Of note, three of these patients showed an upward trend in MDW during the course of their illness. However, the increases in MDW corresponded with clinical deterioration and need for escalation of care, with worsening hypoxia, increased work of breathing, and/or radiologic progression of pulmonary infiltrates. This cohort represents a small subgroup of the study population and therefore larger studies are needed to assess whether MDW corresponds with clinical response over time.

### Monocyte anisocytosis confers greater insight into COVID-19 severity in children than other hematologic and inflammatory biomarkers

**Figure 3:**
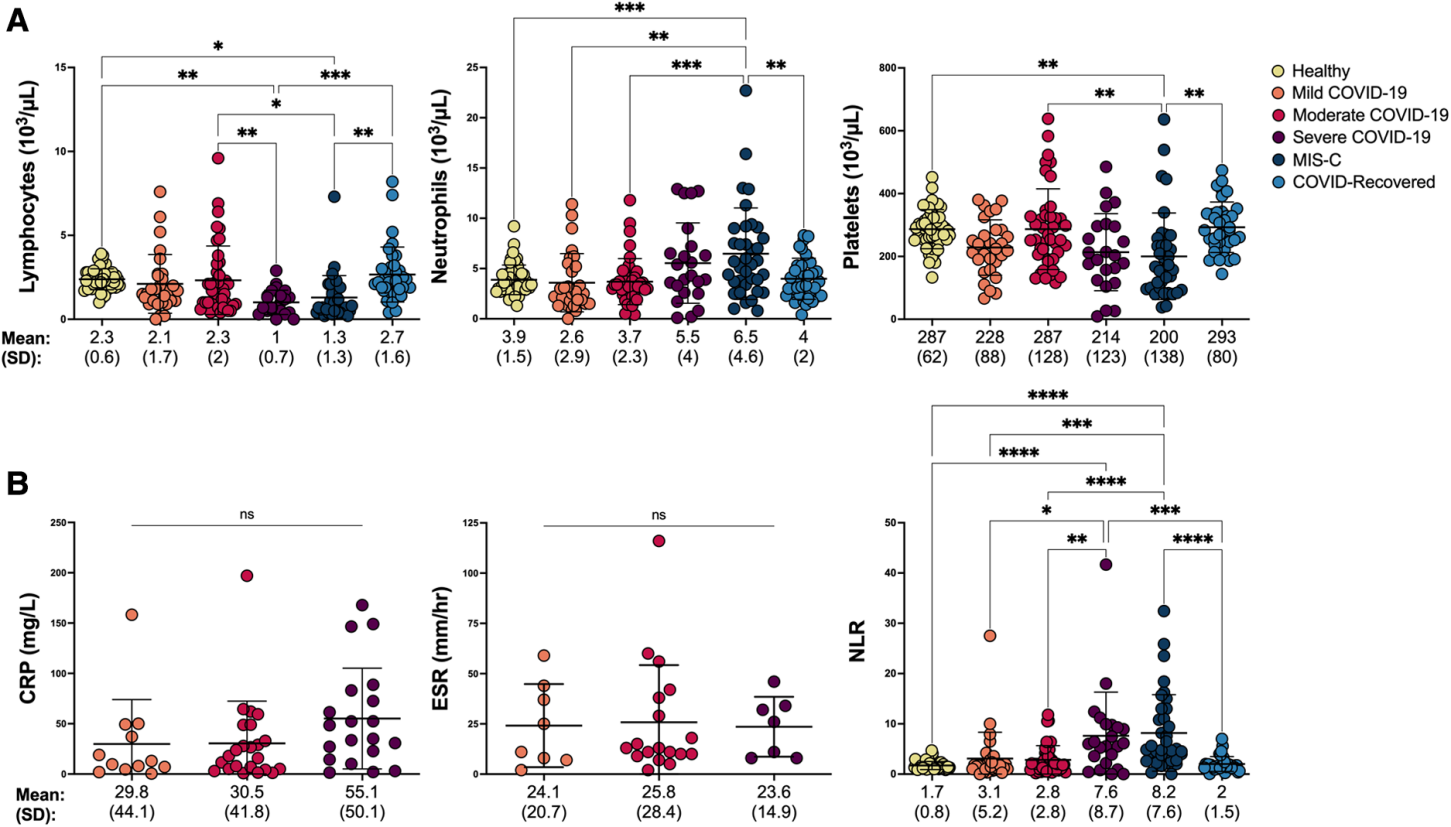
Hematologic and inflammatory parameters in pediatric SARS-CoV-2 infection shift with COVID-19 severity. (**A**) Hematologic parameters, including absolute lymphocyte, neutrophil, and platelet counts were compared between healthy controls, children with mild, moderate and severe COVID-19, MIS-C and asymptomatic children who recovered from COVID-19 by one-way ANOVA and Tukey’s multiple comparisons test. ****p < 0.0001, ***p < 0.001, **p < 0.01, *p < 0.05. (**B**) Inflammatory markers including C-reactive protein (CRP) and erythrocyte sedimentation rate (ESR) were compared by one-way ANOVA when collected within 72 h of MDW in children with COVID-19 (Mild COVID-19: CRP *n* = 12, ESR *n* = 8; Moderate COVID-19: CRP *n* = 23, ESR *n* = 18; Severe COVID-19: CRP *n* = 20; ESR *n* = 7). Neutrophil/lymphocyte ratio (NLR) was analyzed by Kruskal Wallis and Dunn’s multiple comparisons test in all healthy controls, children COVID-19, MIS-C and asymptomatic children who recovered from COVID-19. ****p < 0.0001, ***p < 0.001, **p < 0.01, *p < 0.05. ns = not significant.

To assess the degree to which other hematologic parameters would be useful in distinguishing COVID-19 severity in children, we obtained absolute lymphocyte, neutrophil, and platelet counts from the same CBC that provided MDW. As expected, lymphopenia was detected in children with severe COVID-19 (24), and neutrophilia and thrombocytopenia were identified in children with MIS-C (25, 26) (Figure 3A). However, neither lymphocyte, neutrophil nor platelet counts could distinguish between children with COVID-19 who required hospitalization from those who did not (Supplementary Figure S2A), suggesting that increased monocyte anisocytosis may offer added insight into severity of SARS-CoV-2 infection.

We also tested whether parameters of systemic inflammation, including CRP, ESR, and neutrophil-lymphocyte ratio (NLR) could aid in identifying SARS-CoV-2 infection severity in children. NLR was calculated from the hematology profile corresponding with MDW, whereas CRP and ESR were extracted from the medical record when collected within 72 h of the hematology profile. Interestingly, while CRP and ESR were unable to distinguish between WHO disease severity (Figure 3B), CRP was significantly elevated in children who required hospitalization (Supplementary Figure S2B, unpaired *t*-test *p *< 0.05). NLR, which has been used to assess systemic inflammation in a variety of disorders, including sepsis (27–30), significantly increased in parallel with COVID-19 severity and development of MIS-C (Figure 3B), but was unable to distinguish COVID-19 assessed based on hospitalization status (Supplementary Figure S2B). Of note, CRP and ESR were not obtained from healthy controls or COVID-recovered individuals, and the inability to detect a statisfically significant difference despite an upward trend in CRP across COVID-19 severity may be a result of limited sample size. Other inflammatory parameters that have been tested for COVID-19 severity and mortality risk assessment in adults such as IL-6, LDH, and procalcitonin (31) were not routinely ordered across our pediatric institutions and therefore not included in analysis.

**Figure 4:**
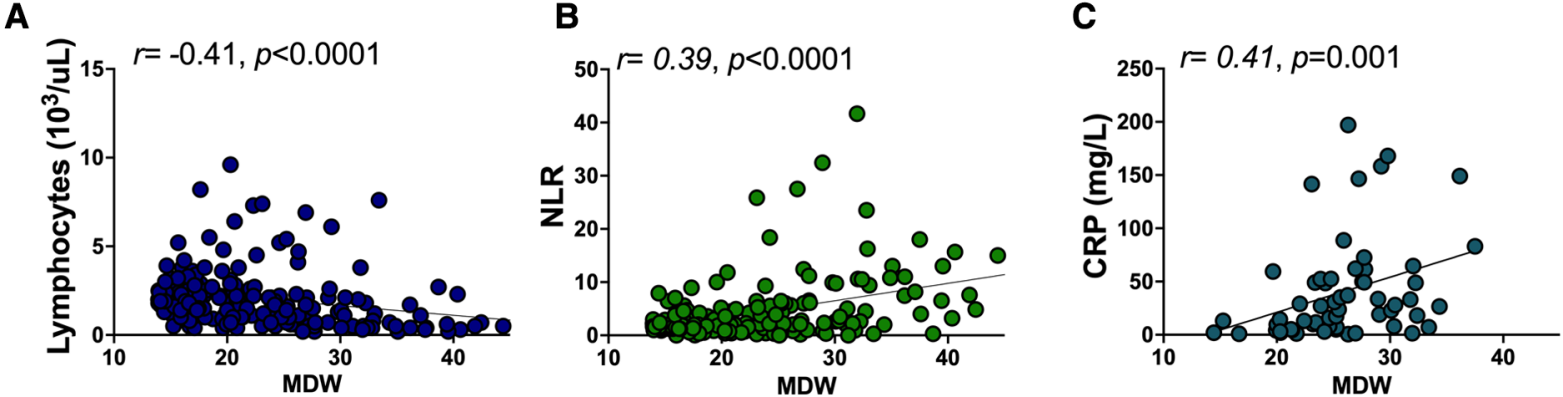
Monocyte distribution width correlates with other hematologic inflammatory parameters. Rank correlation between (**A**) absolute lymphocyte count, (**B**) NLR, and (**C**) CRP and MDW were obtained using Spearman’s rank correlation coefficient.

Since select hematologic parameters captured COVID-19 severity or the need for hospitalization, we then performed regression analysis to determine which parameters correlated with MDW to provide insight into inflammatory activation associated with increasing monocyte anisocytosis. Decreasing absolute lymphocyte count, and increasing neutrophil/lymphocyte ratio and CRP displayed the strongest correlation with increasing MDW (Figure 4, Spearman correlation, *p *< 0.0001, *p *< 0.0001, and *p *= 0.001, respectively) suggesting a broad but interconnected shift of the hematologic profile in response to systemic inflammation.

**Figure 5:**
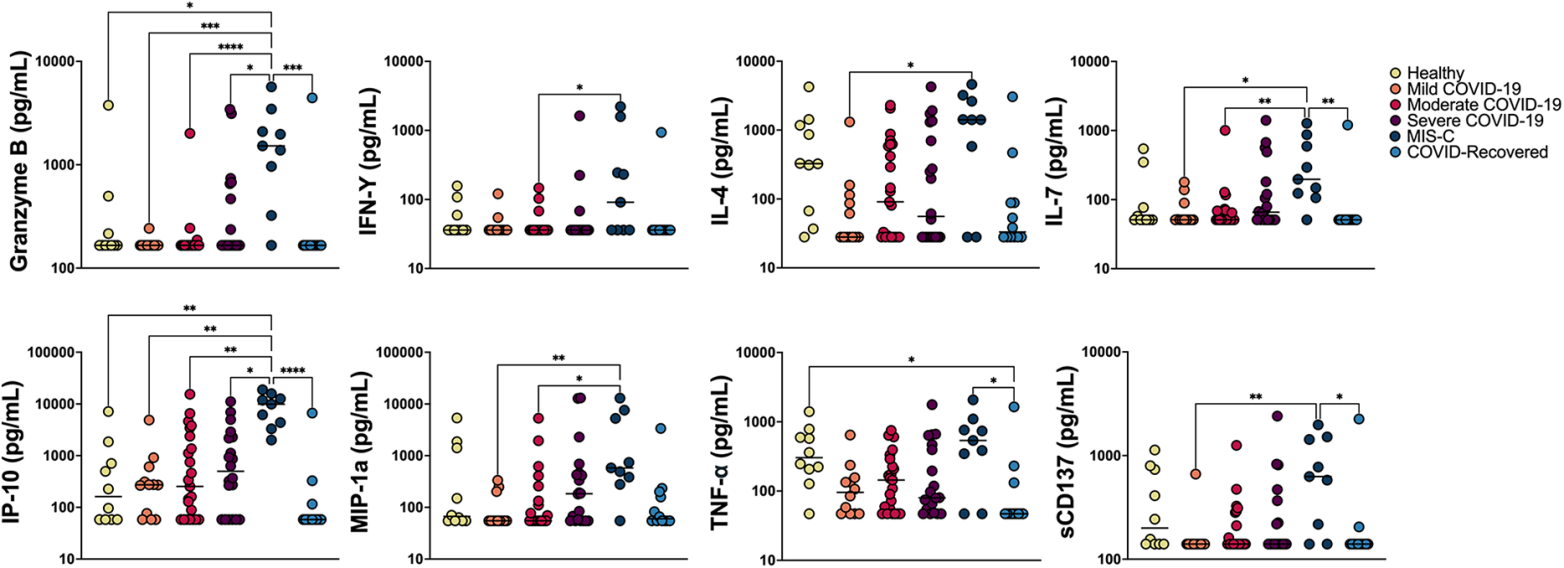
Multiplex cytokine analysis. Multiplex cytokine profiling was obtained from a total of 86 patients: COVID-19 (*n* = 12 mild, 23 moderate, 20 severe), MIS-C (*n* = 9), COVID-19 recovered (*n* = 12) and healthy age-matched controls (*n* = 10). Cytokine concentration (raw data) was analyzed across cohorts using Kruskal-Wallis and Dunn’s multiple comparisons test and plotted on a log scale. Negligible cytokine values reported below the level of detection were set at the lower level of detection for analysis. ****p < 0.0001, ***p < 0.001, **p < 0.01, *p < 0.05.

In order to further characterize underlying immune signaling correlated with severe pediatric COVID-19 and understand immune signatures associated with monocyte anisocytosis, we performed multiplex cytokine profiling on 86 patients across the following cohorts: COVID-19 (*n* = 12 mild, 23 moderate, 20 severe), MIS-C (*n* = 9), COVID-19-recovered (*n* = 12) and healthy pediatric controls (*n* = 10). All samples used for analysis were date- and time-matched with complete blood count. While many cytokines were significantly elevated in MIS-C, (Granzyme B, INF- γ, IL-4, IL-7, IP-10, MIP-1α, TNF-α, sCD137) Figure 5, Supplementary Figure S3), consistent in part with prior immunoprofiling reports (32, 33), these cytokines were unable to distinguish between WHO severity cohorts of pediatric COVID-19.

### Immune signatures incorporating monocyte anisocytosis effectively capture COVID-19 severity in children

**Figure 6:**
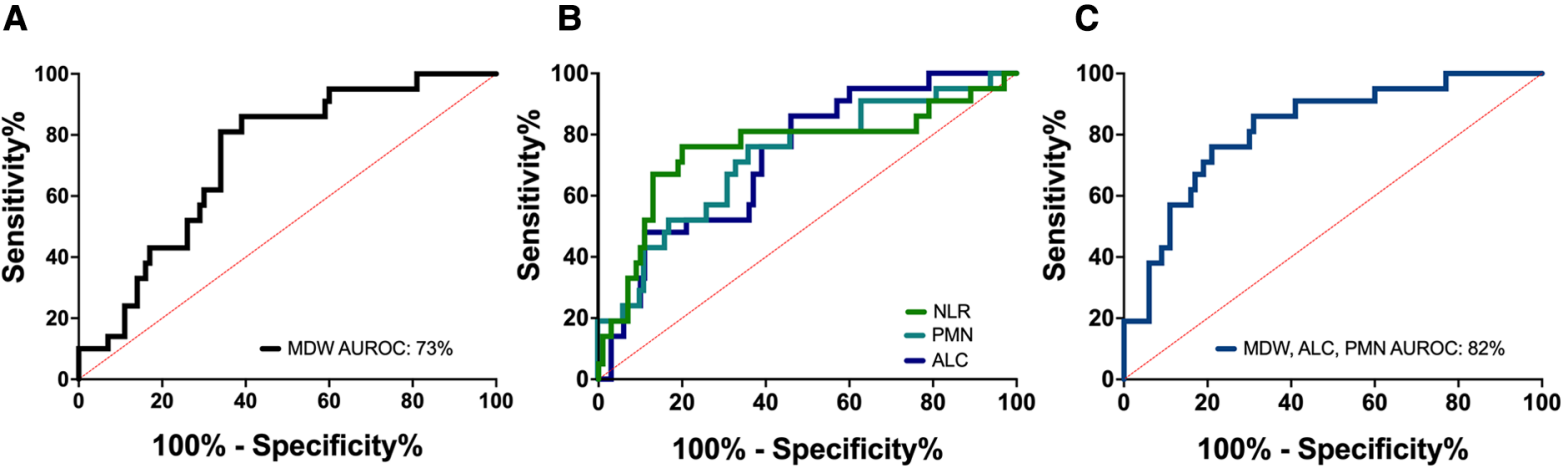
Monocyte distribution width can be assessed in combination with other parameters to more accurately distinguish between COVID-19 severity. (**A**) Receiver operator curve (ROC) of MDW in children with severe vs. non-severe COVID-19. Area under the receiver operator curve (AUROC) was 72.5%. MDW threshold of 23 could distinguish children with severe and non-severe COVID-19 with 85.7% sensitivity and 61.4% specificity (95% CI 61.3 to 83.7%). (**B**) ROC of absolute neutrophil count (AUROC 71.6%), absolute lymphocyte count (AUROC 71.5%), and neutrophil/lymphocyte ratio (AUROC 75.1%). (**C**) ROC for the combined assessment of MDW, absolute neutrophil and lymphocyte count (AUROC 82%).

Lastly, we tested whether the hematologic changes that were determined to be significant could distinguish between severe (*n* = 21) and non-severe (ie. mild and moderate, *n* = 42) cases of COVID-19 in children. We analyzed a receiver operator curve (ROC) of MDW, which alone had an AUROC of 72.5%, and determined that an MDW cut-off value of 23 displayed 85.7% sensitivity and 61.4% specificity for identifying severe cases of pediatric COVID-19 (95% CI 61.3 to 83.7%) (Figure 6A). In our cohort, only three children with mild/moderate COVID-19 had an MDW below 23, whereas 20 children with severe COVID-19 had an MDW over this threshold (Figure 1). Similarly, when comparing severe COVID-19 with mild/moderate COVID-19 in our cohort, ROC for absolute neutrophil count and ROC for lymphocyte counts showed AUROC of 71.6% and 71.5%, respectively, whereas NLR showed an AUROC of 75.1% (Figure 6B, Table 3).

**Table 3:** Diagnostic accuracy of hematologic biomarkers.

Biomarker	AUROC, %	95% CI
MDW + ALC + PMN	82	72–92
MDW + NLR	80.1	70.4–89.9
MDW + ALC	79.3	68.9–89.6
Neutrophil Lymphocyte Ratio	75.1	61.2–89.1
MDW	72.5	61.3–83.7
Absolute neutrophil count	71.6	58.5–84.6
Absolute lymphocyte count	71.5	60–83.1

Area under the Reciever Operator Curve (AUROC) is shown with 95% confidence interval (CI) for hematologic parameters, used alone and in combination, for differenciating between severe and non-severe (mild/moderate) COVID-19 in children. MDW, monocyte distribution width; ALC, absolute lymphocyte count; PMN, polymorphonuclear neutrophil; NLR, neutrophil lymphocyte ratio.

Thus, we tested whether linear combinations of MDW, absolute neutrophil and lymphocyte counts, and NLR could provide greater diagnostic accuracy for severe pediatric COVID-19. We found that calculated indexes combining MDW and lymphocyte count or MDW and NLR displayed an AUROC of 79.3% and 80.1%, respectively, which suggests that these biomarkers can more appropriately capture the inflammatory signature that distinguishes severe pediatric COVID-19 when used in combination (Table 3). The highest AUROC was 82.0% for an index that combined MDW, neutrophils, and lymphocytes (Figure 6C, Table 3). The overall effectiveness of this index was estimated at 54.3% using Youden’s J statistic. Importantly, the power of this combination increased when assessed prior to treatment with corticosteroids in a subset of 70 pediatric patients with COVID-19 (AUROC 83.3%; CI 71.9% to 94.8%; Youden index 0.698). Altogether, these findings indicate that a combined assessment of hematologic parameters, including detection of monocyte anisocytosis, can be a useful tool for identifying severe COVID-19 in children.

## Discussion

Although the vast majority of children with SARS-CoV-2 infection develop mild symptoms, progression to severe disease requiring hospitalization can occur as well. However, the utility of biomarkers for assessment of COVID-19 severity is not as well established in the pediatric population as compared to adults. Here, we examined monocyte anisocytosis in a multicenter cohort of children infected with SARS-CoV-2 and identified an increase in MDW associated with pediatric SARS-CoV-2 infection. Thus, our findings indicate that MDW, which can be obtained from a standard complete blood count to quantify monocyte anisocytosis, may serve as a clinically useful tool in the evaluation of children with COVID-19.

Importantly, we found that MDW greater than 23 was associated with severe COVID-19 in children within our cohort, and that monocyte anisocytosis corresponded with shifting hematologic profiles and increasing general inflammatory markers signaling infection-mediated immune activation. When taking into consideration other inflammatory markers, such as NLR, the sensitivity and specificity for distinguishing severity of COVID-19 increased even further. Due to the overlap in MDW values across the spectrum of COVID-19, monocyte anisocytosis should not be used alone to determine disease severity in SARS-CoV-2 infected children, but rather evaluated in combination with clinical presentation. Interestingly, children hospitalized for COVID-19 had significantly higher MDW compared to that of non-hospitalized patients, despite longer time from SARS-CoV-2 detection to sample collection, suggesting an exaggerated innate immune activation in the setting of waning viral load (34) in more severe pediatric disease, reminiscent to that seen in adults (3). Thus, quantification of monocyte anisocytosis provides important, clinically relevant insight into the inflammatory response to SARS-CoV-2 infection in children.

Extensive immune profiling of SARS-CoV-2 infection has established that monocytes are key mediators of disease severity in both children and adults (9), supporting the utility of monocyte-specific markers of immune activation in COVID-19. In severe COVID-19, inflammatory monocyte signatures predominate and delayed or impaired type I interferon (IFN) production contribute to increased levels of IL-6 and subsequent cytokine storm (35, 36). In contrast, immune responses in patients with mild disease are characterized by rapid-onset type I IFN production with subsequent induction of interferon-stimulated genes, ultimately resulting in the inhibition of viral replication (37). While the impact of IFN signaling on monocyte anisocytosis has not been described, others have suggested that increased MDW reflects inflammasome activation or myeloid suppression as a result of viral infection (38). Although we did not define mechanisms driving monocytes anisocytosis in this study, we identified phenotypic differences in monocyte population across disease severity which warrant further exploration and may provide additional insight into disease pathogenesis (3, 36).

MDW has previously been established as a marker of sepsis in adults (15) and described in multiple other disease states, including sepsis in children (12), adult COVID-19 (17, 23, 39) and MIS-C (18). However, the stark dichotomy in innate immune responses to SARS-CoV-2 infection between children and unvaccinated adults, particularly pertaining to monocytes (2, 3), demanded that monocyte anisocytosis be assessed specifically within a pediatric population. Children are capable of mounting a robust immune response to SARS-CoV-2 with an earlier clinical resolution of inflammation than what is typically seen in adults (2, 3). Additionally, studies have shown that while children display reductions in all monocyte subsets during acute infection, adults display an expanded classical (CD 14^+^ CD 16^−^) and intermediate (CD 14^+^ CD 16^+^) monocyte signature (2). Despite these differences, we found that monocyte anisocytosis is effective at capturing distinctions in disease severity in pediatric COVID-19, similar to what has been described in adults (3, 39).

Our study had limitations: While we enrolled nearly 900 children in our multicenter study, only 94 children were acutely infected with SARS-CoV-2, which may speak to the low rates at which SARS-CoV-2-infected children required medical attention. Additionally, we were only able to collect sequential blood draws, analyze cytokine profiles, and obtain other inflammatory parameters on a subset of this pediatric cohort. Thus, larger studies are needed to inform utility of tracking monocyte anisocytosis over a hospital course. Clinical considerations including the impact of vaccination and variants of concern on monocytic responses also need to be defined in the future. Additionally, the role of MDW as a general immune marker needs to be further explored in cohorts with other viral infections and compared with SARS-CoV-2-related illness. A rise in MDW in the setting of acute disease is non-specific to SARS-CoV-2 infection and the presence of monocyte anisocytosis does not preclude the need for antibiotic treatment in patients if clinically indicated.

In conclusion, as COVID-19 resulted in significant morbidity and mortality in children during the height of the pandemic (6), biomarkers are needed to identify those at risk in the setting of future COVID-19 endemic or pandemic outbreaks. Monocyte anisocytosis is helpful for identifying patients with severe COVID-19, given that it provides a window into the underlying monocyte-driven immune response to SARS-CoV-2 infection and corresponds with clinical severity and need for hospitalization.

## Data Availability

The raw data supporting the conclusions of this article will be made available by the authors upon reasonable request.

## Supplementary material

SUPPLEMENTARY FIGURE S1

Changes in MDW over time. Compared to initial MDW value (day 0), changes in MDW were grouped by day from initial MDW assessment. Analysis by one-way ANOVA (*p = 0.0004)*.

SUPPLEMENTARY FIGURE S2

Hematologic and inflammatory parameters in pediatric SARS-CoV-2 infection shift with COVID-19 severity, but only CRP captures the need for hospitalization. (**A**) Hematologic parameters from patients with COVID-19 were analyzed based on hospitalization status (outpatient, *n* = 42; hospitalized *n* = 52) using unpaired t-test. (**B**) Inflammatory markers were analyzed in patients with COVID-19 based on hospitalization status (Outpatient: CRP *n* = 15, ESR *n* = 10, NLR *n* = 42; Hospitalized: CRP *n* = 40, ESR *n* = 23, NLR *n* = 52). CRP and ESR were analyzed using unpaired t-test, and analysis of NLR was done using Mann-Whitney U test. **p < *0.05, ns = not significant.

SUPPLEMENTARY FIGURE S3

Multiplex cytokine analysis. Cytokine data was obtained from a total of 86 patients: COVID-19 (*n* = 12 mild, 23 moderate, 20 severe), MIS-C (*n* = 9), COVID-19 recovered (*n* = 12) and healthy age-matched controls (*n* = 10). Cytokine concentration (raw data) was analyzed across cohorts using Kruskal-Wallis and Dunn’s multiple comparisons test and plotted on a log scale. Negligible cytokine values reported below the level of detection were set at the lower level of detection for analysis. Non-significant comparisons are not displayed.

SUPPLEMENTARY TABLE S1

Clinical features of acute pediatric SARS-CoV-2 infection. Clinical features of pediatric patients with non-severe (mild/moderate) and severe COVID-19. Of note, the seven children with non-severe COVID-19 maintained documented oxygen saturations greater than 90% for the duration of their illness, including prior to oxygen supplementation.

SUPPLEMENTARY TABLE S2

Mean and standard deviation of biomarkers across cohorts.
