# Supplementary figures and images for: Monocyte anisocytosis corresponds with increasing severity of COVID-19 in children
Source: Front Pediatr. 2023 Jun 23;11:1177048. doi: 10.3389/fped.2023.1177048 (PMC10326545; doi:10.3389/fped.2023.1177048)

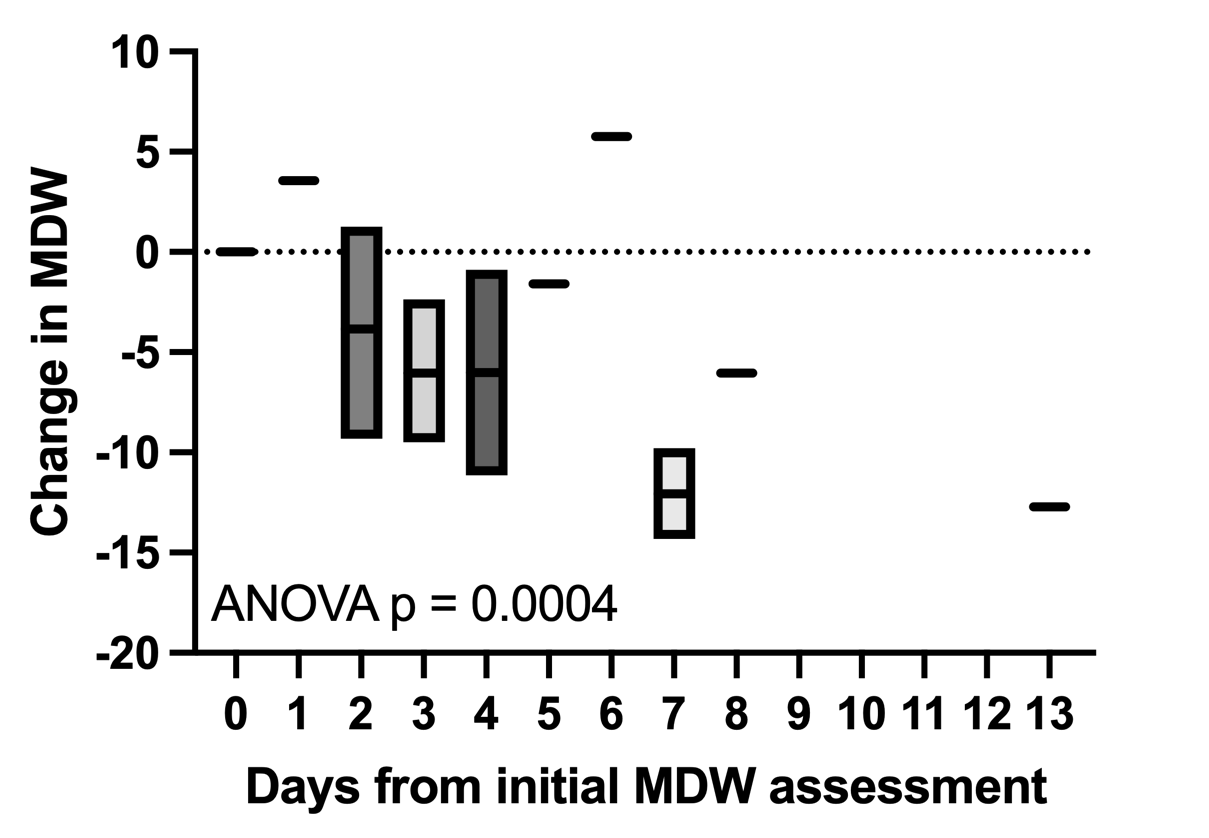

Supplement: Supplementary file 3 [file Image1.tiff]

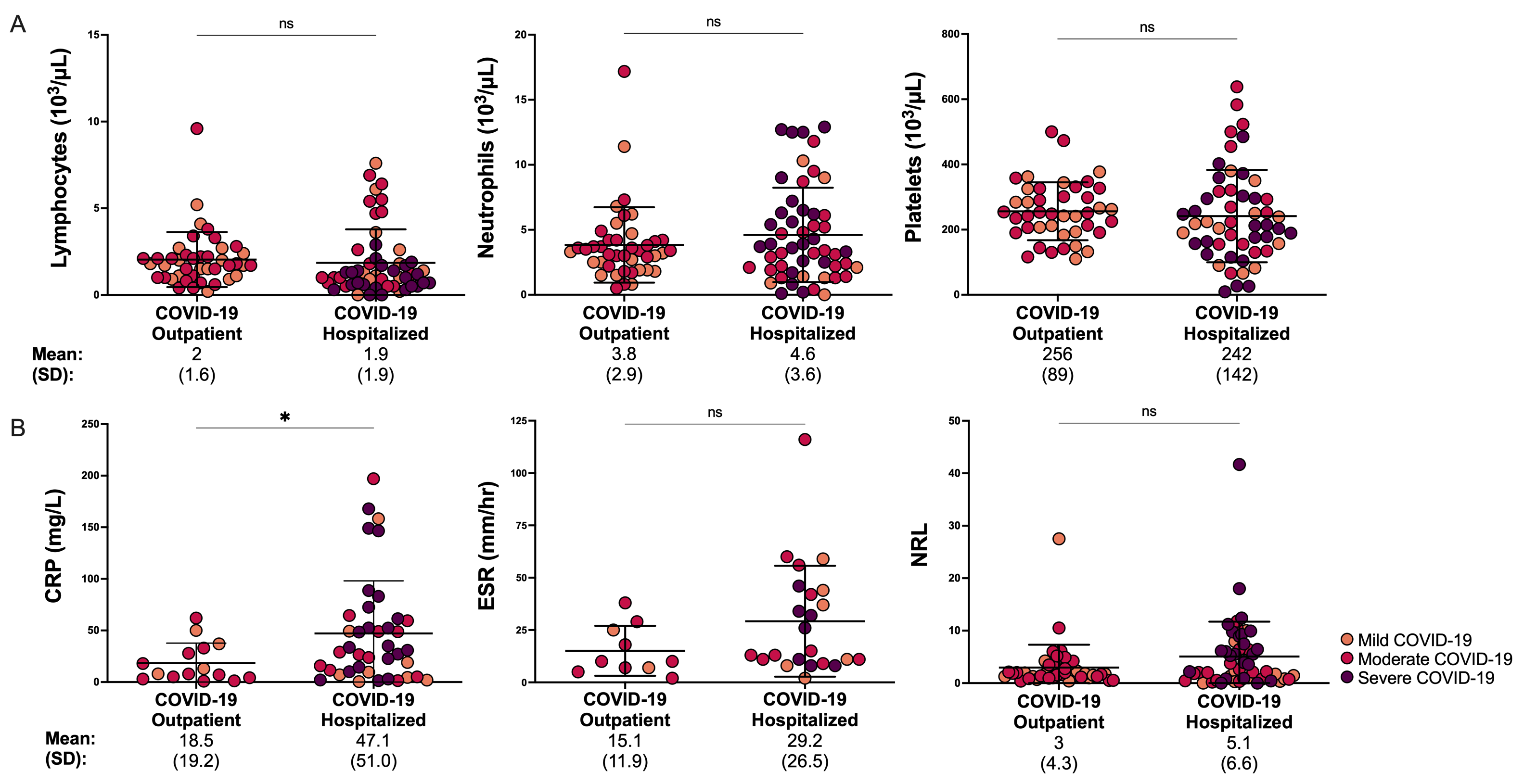

Supplement: Supplementary file 4 [file Image2.tiff]

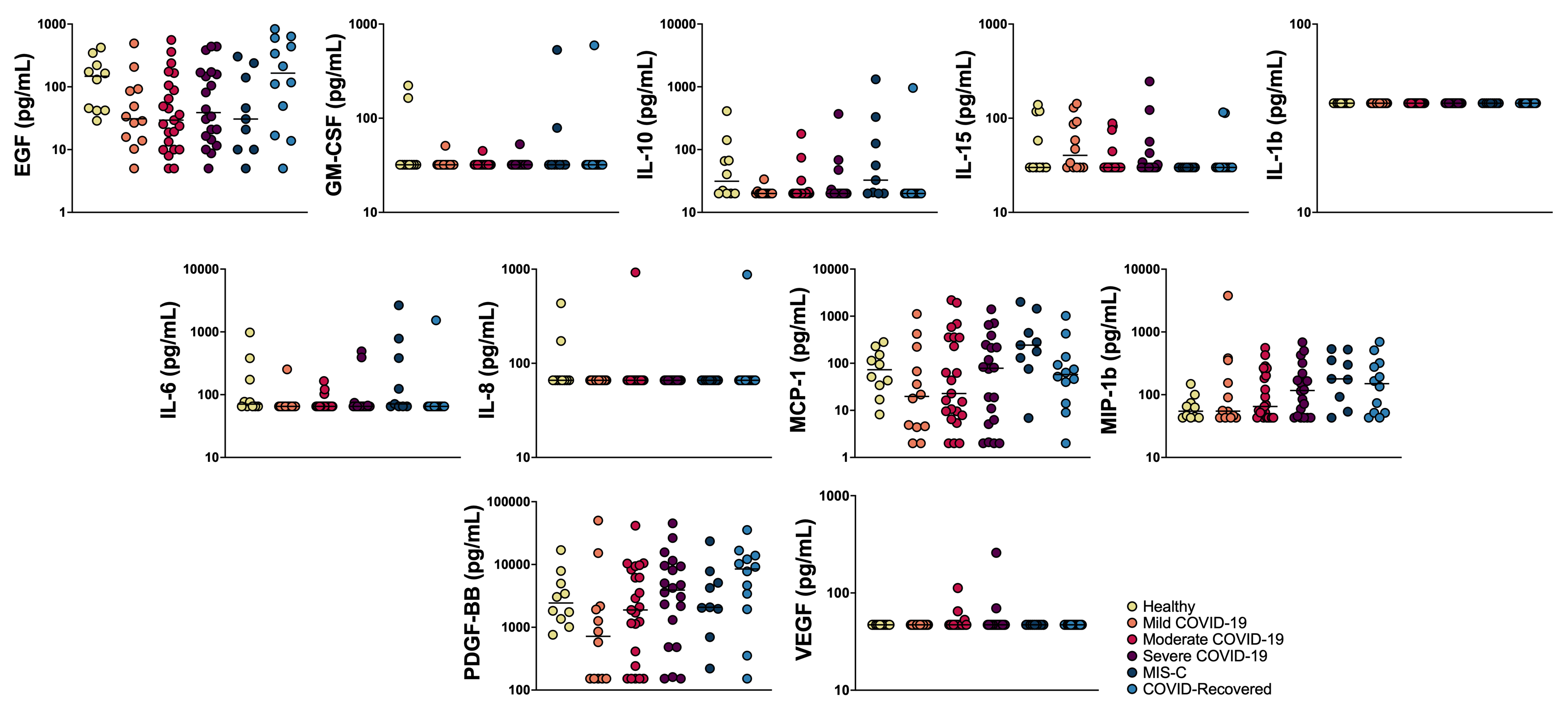

Supplement: Supplementary file 5 [file Image3.tiff]
